# Anti-Obesity Effect of *Nostoc commune* Ethanol Extract In Vitro and In Vivo

**DOI:** 10.3390/nu14050968

**Published:** 2022-02-24

**Authors:** Sheng-Chieh Tsai, Yu-Wen Huang, Chih-Chung Wu, Jyh-Jye Wang, Ya-Ting Chen, Reeta Rani Singhania, Chiu-Wen Chen, Cheng-Di Dong, Shu-Ling Hsieh

**Affiliations:** 1 Department of Seafood Science, National Kaohsiung University of Science and Technology, Kaohsiung 81157, Taiwan; b220682837@gmail.com (S.-C.T.); may2377234@gmail.com (Y.-W.H.); melodyyu.chen@gmail.com (Y.-T.C.); 2 Department of Medical Research, E-Da Hospital, Kaohsiung 82445, Taiwan; 3 Department of Food and Nutrition, Providence University, Taichung 43301, Taiwan; wuccmail@gmail.com; 4 Department of Nutrition and Health Science, Fooyin University, Kaohsiung 83102, Taiwan; ft054@fy.edu.tw; 5 Department of Marine Environmental Engineering, National Kaohsiung University of Science and Technology, Kaohsiung 81157, Taiwan; reetasinghania@nkust.edu.tw (R.R.S.); cwchen@nkust.edu.tw (C.-W.C.); cddong@nkust.edu.tw (C.-D.D.)

**Keywords:** *Nostoc commune*, phytochemical, anti-oxidations ability, 3T3-L1 pre-adipocytes, obesity

## Abstract

*Nostoc commune* is an edible terrestrial blue-green alga. It has shown many beneficial effects on human health. This study aimed to investigate the phytochemical assay of *N. commune* ethanol extract (NEE) and its anti-obesity effects. The effect of a high-calorie diet on lipid accumulation in 3T3-L1 preadipocytes is investigated, and a Wistar rat model is used to demonstrate the anti-obesity effect of NEE and its mechanism. The results showed that the NEE has phytochemical compounds, such as total polyphenol, total flavonoids, and total terpenoids. NEE was also shown to suppress cell proliferation and lipid accumulation (26.9%) in 3T3-L1 preadipocytes. Furthermore, NEE reduced the body weight (13.5%), fat tissue weight (13.3%), and the serum FFA (19.4%), TG (14.2%), TC (11.8%), and LDL-C (16.4%) of rats. In histopathology, NEE was shown to diminish the size of adipocytes and hepatic lipid droplets. The NEE downregulated the mRNA expression of adipogenesis (PPAR-γ, SREBP-1c) and lipid lysis-related genes (ATGL, HSL) in epididymal adipose tissue. The NEE also upregulated the mRNA expression of β-oxidation related genes (AMPK, CPT-1, PPAR-α) in the liver. Overall, this study suggests NEE has the potential to be developed as a functional food for anti-obesity.

## 1. Introduction

Obesity is defined as an excess of fatty tissue in the body, which increases the risk of a variety of chronic illnesses and body function disorders, such as diabetes mellitus type 2, hypertension, cardiovascular disease, and obese sarcopenia. [1,2]. Adipocyte size and quantity increase, resulting in a low-grade pro-inflammatory milieu in adipose tissue, which leads to fat gain [3]. It is important to study the role of fat in obesity for the prevention as well as treatment of obesity. Lipogenesis and lipolysis are important processes for the control of adipocyte energy balance. Peroxisome proliferator-activated receptor γ (PPARγ) is one of the transcription factors that is important in the transformation of preadipocytes into mature adipocytes; sterol regulatory element-binding protein (SREBP) expression helps triglyceride synthesis in adipocytes, which leads to adipocyte lipid accumulation [4,5]. In addition, a decrease in adipocyte size is an effective therapy for obesity. Adenosine monophosphate-activated protein kinase (AMPK) is a key factor of energy homeostasis, because it is a fuel-sensing enzyme that is activated by an increased ratio of AMP/ATP and is sensitive to nutrition conditions [6,7]. Hormone-sensitive lipase (HSL) and adipose triglyceride lipase (ATGL) promote lipolysis and lyse triglyceride into monoglyceride and free fatty acid in adipocyte lipid droplets, with an increase of up to 95%, and are the key factors of lipolysis. [8,9,10]. Peroxisome proliferator-activated receptor α (PPAR-α), present in adipocyte, hepatocyte, muscle, and epithelial cells, acts in controlling lipid and glucose homeostasis [11,12,13]. Its activation increases mitochondria β-oxidation-promoting fatty acid catabolism, which results in lipid content reduction. Carnitine palmitoyltransferase I (CPT-1) inhibits malonyl-CoA to reduce lipogenesis, controlling the β-oxidation process of long-chain fatty acid into mitochondria [14,15]. For the management of improving obesity, a change in lifestyle, eating healthy food, and an increase in physical activity are necessary, but few individuals can maintain these healthy habits for a long period. Therefore, the development of new weight-loss drugs is important in the study of anti-obesity. Recently, interest in using algae as a dietary or functional food has increased tremendously because it contains rich vitamins and an abundance of bioactive compounds [1,16]. Many studies on anti-obesity using seaweed extract or seaweed derivatives for cell or animal models have been conducted, but rarely for terrestrial algae.

*Nostoc commune* (*N. commune*) has been found in tropical soil, temperate zones, and both the northern and southern polar zones. In freshwater settings, it frequently develops visibly extensive mucilaginous layer colonies on soil and is typically observed on stones and dirt. A macroscopic cyanobacterium known as *N. commune Vaucher* is used as a nutritious food and traditional medicine all over the world [17]. Anti-infectious and anti-bacterial activity [18,19,20,21], anti-oxidative capabilities, anti-cancer activity, immunomodulation, and cholesterol reduction [22,23,24,25,26,27,28,29,30,31,32] have all been demonstrated in *N. commune* or *N. commune* derivatives. A previous study showed that the *N. commune* lipid extract reduced cholesterol synthesis and fatty acid metabolism through involved protein expression of 3-hydroxy-3-methylglutaryl-CoA reductase (HMGR) and reduced gene expression of SREBP-1. However, few studies are available on its anti-obesity and lipid metabolism–regulating abilities.

Therefore, we aimed to study *N. commune*’s anti-obesity functions and clarify its molecular mechanisms. This study identifies *N. commune* ethanol extract (NEE) as having three phytochemicals, and we determined total polyphenols, total flavonoids, and total terpenoids. We also investigated the effect of NEE on cell proliferation and lipid accumulation in 3T3-L1 preadipocytes.

Finally, in a high calorie diet–induced obese Wistar rat, NEE was examined to determine whether lipolysis and lipogenesis gene expression influenced body weight, serum parameters, and tissue histology.

## 2. Materials and Methods

### 2.1. Chemicals and Reagents

Ethanol (95%) was purchased from Taiwan Tobacco & Liquor Corporation (Taipei, Taiwan). Aluminum chloride was purchased from Kanto Chemical (Tokyo, Japan). Rutin was purchased from Chromadex (Los Angeles, CA, USA). Folin-Ciocalteu 2N, sodium carbonate, gallic acid, sodium nitrite, sodium hydroxide, 3-isobutyl-methylxanthine (IBMX), dexamethasone (DEXA), insulin, oil red O, triton X-100, formalin, TRIzol reagent, 3-(4,5-dimethyazol-2-yl)-2,5-diphenyltetrazolium bromide (MTT), sodium bicarbonate, and chloroform was purchased from Sigma-Aldrich (St. Louis, MO, USA). Isopropanol was purchased from J.T. Baker (Pittsburgh, PA, USA), and 10x phosphate-buffered saline was purchased from Uni-onward (Taipei, Taiwan). Penicillin/streptomycin, fetal bovine serum (FBS), Dulbecco’s modified eagle high-glucose medium (DMEM), and trypsin were purchased from Gibco (New York, NY, USA). RANDOX produced the commercial kits for measuring total triglycerides (TR212), total cholesterol (CH202), high-density lipoprotein-cholesterol (CH2655), low-density lipoprotein-cholesterol (CH2657), lipase activity (LI118), and ketone body (RB1007) (Ireland, UK). Abcam produced the commercial kits for measuring free fatty acid (ab65341), glucose (ab65333), alanine transaminase activity (ab105134), aspartate aminotransferase activity (ab105135), blood urea nitrogen (ab83362), and creatinine (ab65340) (Cambridge, UK). Promega produced the RNase, Oligo dT, and M-MLV reverse transcriptase (Madison, WI, USA). Topgen produced DEPC water, SYBR fast mix (2×), and primer (Kaohsiung, Taiwan).

### 2.2. Sample Preparation

*N. commune* was purchased from a local market (Pingtung, Taiwan). It was washed twice with distilled water, dried at 50 °C to a water content of less than 10%, and then crushed into powder with a pulverizer to convert it into *N. commune* powder. *N. commune* powder (100 g) was mixed with 300 mL of 95% alcohol (1:3 *w*/*v*) at room temperature for 1 h. The extract (*N. commune* ethanol extract, NEE) was filtered and concentrated by rotary evaporation using a speed vacuum. Afterward, the concentrated sample was freeze-dried and stored at −20 °C (final extraction yield of 7.1%). All NEE samples were sonicated (Ultrasonic Cleaner, DC200) until resuspended in ethanol or PBS for future experiments.

### 2.3. Analysis of Phytochemicals

#### 2.3.1. Total Polyphenol Quantitative Method

Total phenolic content was measured by the Folin-Ciocalteau method [33]. In brief, dry 0.1 mg NEE and 0.1 mL ethanol (95%) were first mixed in a 1.5 mL tube, and then 0.1 mL Folin reagent (1N) was added and vortexed. After 5 min standing in the dark, 0.5 mL sodium carbonate solution (7.5%) was added and vortexed. In the dark, the mixture was incubated for 30 min at room temperature. The absorbance was measured spectrophotometrically at 760 nm using a UV-vis spectrophotometer (Bio-Tek, Winooski, VT, USA) against a solvent blank of ethanol. A gallic acid (GAE) solution was used as a standard for calculation, and total phenolic content was expressed as GAE µg/NEE mg.

#### 2.3.2. Total Flavonoid Quantitative Method

The flavonoid content method was quantified in accordance with Layzon [34]. In brief, dry 0.1 mg NEE and 0.1 mL ethanol (95%) were mixed in a 1.5 mL tube. The 0.1 mL fresh sodium nitrite solution (5%) and 0.1 mL aluminum chloride solution (10%) were added to the mixture and incubated for 6 min. The mixture was added to 0.6 mL of sodium hydroxide solution (10%) and incubated for 30 min at room temperature. Samples were measured calorimetrically at 510 nm using a UV-vis spectrophotometer (Bio-Tek, Winooski, VT, USA) having ethanol as a blank. A Rutin (RUT) solution was used as a standard for calculation, and total phenolic content was expressed as RUT µg/NEE mg.

#### 2.3.3. Total Terpenoid Quantitative Method

Total terpenoid content was determined using acetic acid-Vanillin/perchloric acid reagent [35]. In brief, 0.1 mg dry NEE and 0.1 mL ethanol (95%) were first mixed in a 2 mL tube. Then, 0.2 mL freshly prepared vanillin in acetic acid solution (5%) and 0.8 mL perchloric acid were added. The mixture was incubated for 15 min in a 70 °C water bath. Then, it was cooled on ice and thoroughly shaken; the absorbance of the samples was measured at 548 nm using a UV-vis spectrophotometer (Bio-Tek, Winooski, VT, USA), and the ethanol as a blank. Oleanolic acid hydrate (OAH) solution was used as a standard for calculation, and total phenolic content was expressed as OAH µg/NEE mg.

#### 2.3.4. 3T3-L1 Preadipocyte Cell Culture and Differentiation

The 3T3-L1 preadipocytes were obtained from the Food Industry Development Institute (Hsinchu, Taiwan) and cultured in DMEM containing 10% FBS, 3.7 g/L sodium bicarbonate, and 100 U/mL penicillin-streptomycin in a humidified atmosphere of 5% CO_2_ incubator at 37 °C. Then, 3T3-L1 preadipocytes were seeded into 5 × 10^4^ cells into a 35 mm dish for 24 h (we defined this as the initial day) and then treated with 50 μg/mL NEE for 10 days. They were differentiated with induction medium (0.5 mM IBMX, 1 μM DEXA, and 10 μg/mL insulin) for 2 days (3rd and 4th day). After the induction medium, cells were treated with 10 μg/mL insulin medium for 4 days (5th to 8th day); during this stage, 90% of the 3T3-L1 preadipocytes were transformed into mature adipocytes, and lipid droplets were formed intracellularly. After differentiation, the cell was cultured with DMEM for 2 days (9th and 10th day). The medium was replenished every other day. The 3T3-L1 preadipocytes were added to the inducing medium alone, serving as the induced group. All the data in the cell model experiment came from three separate investigations.

### 2.4. Cell Proliferation Analysis in 3T3-L1 Preadipocytes

The 3T3-L1 preadipocytes were seeded in 5 × 10^4^ cells in a 35 mm dish and allowed to adhere overnight. Afterward, the adhered cells were treated with different concentrations of NEE (5, 10, 25, 50 μg/mL) for 72 h. The experimental cell sample was harvested and diluted to an experimental volume. The cell pellets were resuspended in 1 mL of PBS; 19 μL was taken for use in another Eppendorf, and 1 μL of solution 13 (Acridine orange with 30 µg/mL concentration and DAPI with 100 µg/mL concentration) was added. The NucleoCounter^®^ NC-3000TM fluorescent imaging cytometer provided the analytical procedure methodology.

### 2.5. Cell Viability Analysis in 3T3-L1 Preadipocytes

MTT was used to determine the viability of the cells [5]. The 3T3-L1 preadipocytes were seeded in 5 × 10^4^ cells in a 35 mm dish and incubated for 1 day. For 10 days, cells were fed with an induction medium containing 50 μg/mL NEE. Optical density was measured at 570 nm after incubation at 37 °C with a medium containing 0.1 mg/mL MTT. In comparison to the control, cell viability (percent) was measured as the proportion of cells that survived.

### 2.6. Lipid Accumulation Test in Differentiated 3T3-L1 Preadipocytes

#### 2.6.1. Oil Red O Staining and Quantitative Analysis in Differentiated 3T3-L1 Preadipocytes

Preadipocytes develop intracellular lipid droplets throughout the differentiation process into adipocytes, which may be stained using Oil Red O. When comparing the differentiated adipocyte control to preadipocyte control, lipid droplets were seen. Collected samples were fixed in formalin (10%) for 1 h. Oil Red O stain solution (0.5 mg Oil Red O and 60% isopropanol mixed with sterile H_2_O) was used to stain fixed cells for 1 h. Before being examined using an optical microscope (Olympus, Tokyo, Japan), stained cells were thoroughly washed in distilled water. After adding the isopropanol, the mixture was stirred for 15 min. At 510 nm, the optical density was measured. The quantitative (percent) amount of oil red was calculated as a proportion of the control.

#### 2.6.2. Triglyceride Deposition Test in Differentiated 3T3-L1 Preadipocytes

The experimental cells were washed with PBS twice and harvested in the 1.5 mL tube to assay the level of cellular triglycerides and protein concentration. The collected cell sample was mixed with PBS 70 μL containing 0.5% triton X-100 and sonicated for 30 s (Qsonica, Newtown, CT, USA). The protein concentration was evaluated using the Lowry method [36], and the TG content was assessed using a commercial TG test kit. The triglyceride deposition results were calculated as triglycerides (mg) per cellular protein (mg).

### 2.7. Animal Care and Treatment

All animal care and experimental techniques used in this study were approved by the Institutional Animal Care and Use Committees (IACUC) of the National Kaohsiung University of Science and Technology (Kaohsiung, Taiwan); 0106-AAAP-007 is the IACUC number. Wistar rats (sex: male; age: six weeks old; weight: 250 ± 20 g) were purchased from BioLASCO Taiwan Co., Ltd. (Taipei, Taiwan). Rats were domesticated in a cage with a half-day light and dark cycle and a constant temperature of 22 °C. The rats were randomly separated into 5 groups (*n* = 10 each group, *n* = 50) after acclimating to the facility for seven days. Rats were fed LabDiet 5001, which we defined as a normal diet (ND) (58.0% carbohydrate, 13.5% lipid, 28.5% protein, 3.36 kcal/g). A high-calorie diet (HD) (45.0% carbohydrate, 45.0% lipid, 10.0% protein, 8.48 kcal/g) was modified from the ND and made from sucrose and soybean oil. The HD was combined with NEE 38.5 mg/kg rat (low-dose NEE, LNEE), with NEE 77.0 mg/kg rat (medium-dose NEE, MNEE), and with NEE 192.5 mg/kg rat (high-dose NEE, HNEE) for 12 weeks. The NEE dosage utilized in this investigation was calculated based on the results of a cell model experiment utilizing rats’ blood and body weight. NEE was given 4 times a week by oral administration. The ND and HD groups were given PBS orally. Food consumption and body weight were recorded four times a week during the course of the 12 week study. The feed conversion rate (FCR) was calculated by dividing the total weight gain (g) by diet consumption (g). Organ weight as a percentage of body weight was (organ weight/body weight) 100%. The rats were starved for 12 h before being euthanized using CO_2_. Blood samples were obtained through heart punctures. The blood sample was centrifuged 2438× *g*/10 min at 4 °C and stored at −20 °C for serum lipid profile and serum parameter analyses. Liver and epididymal fat were removed and fixed in 10% formalin or stored at −80 °C for subsequent analyses. The data analysis of growth features and serum biochemical parameters in animal experimental models was done using 10 rats per group.

### 2.8. Animal Serum Biochemical Parameters

According to the manufacturer’s instructions, commercial kits were used to assess TC, TG, LDL-C, HDL-C, glucose, FFA, ketone bodies, lipase active, BUN, creatinine, ALT, and AST levels.

### 2.9. Histopathological Analysis

Formalin (10%) was used to fix epididymal adipose tissue and hepatic tissue, which was then embedded in paraffin and cut into 4 mm thick slices. According to the Harris hematoxylin and eosin staining standard protocol, the sections were stained with hematoxylin and eosin. Under the microscope (Olympus, Tokyo, Japan), the dyed slides were examined.

### 2.10. Real-Time Quantitative Polymerase Chain Reaction (qPCR)

Total RNA was isolated from epididymal adipose tissue and hepatic tissue using TRIzol reagent following the manufacturer’s protocol. Total RNA was measured using an Epoch microvolume spectrophotometer (Bio-Tek, Winooski, VT, USA). After RNase treatment and RNA repurification, the cDNA was synthesized using M-MLV reverse transcriptase. Quantitative PCR analysis was advanced using SYBR green. Real-time PCR (LightCycler^®^ 96 Instrument, Roche Life Science, Switzerland) was used to measure relative gene expression. The amplified reaction conditions were the following for the melting analysis: program 1 (95 °C, 120 s, carried out for 1 cycle), program 2 (95 °C, 5 s, and cooling down to 60 °C, 30 s, carried out for 40 cycles), program 3 (95 °C, 10 s, and cooling down to 65 °C, 60 s, and then heated to 97 °C, 1 s, carried out for 1 cycle). The control group for the gene-specific primers was β-actin, and the target genes were as shown in Table S1. For the mRNA gene expression of lipid lysis and β-oxidation-related gene, every group was selected (*n* = 5) for the independent experiment.

### 2.11. Statistical Analysis

The data were examined using the statistical analysis program SPSS for Windows, version 20.0. (SPSS, Inc., Chicago, IL, USA). A one-way analysis of variance and Duncan’s test were performed to evaluate the significance of differences between two mean values. A statistically significant result was defined (*p* < 0.05). Different superscript letters indicate that they are statistically different from each other.

## 3. Results

### 3.1. Phytochemical Composition in NEE

Quantitative analysis of NEE revealed the presence of major phytochemical compounds, as shown in Table 1. The NEE phytochemical compounds of total polyphenols, total flavonoids, and total terpenoids are 25.89 ± 1.18 GAE µg/NEE mg, 19.32 ± 0.45 RUT µg/NEE mg, and 926.53 ± 0.03 OAH µg/NEE mg.

### 3.2. Effect of NEE on Cell Proliferation in 3T3-L1 Preadipocytes

**Figure 1:**
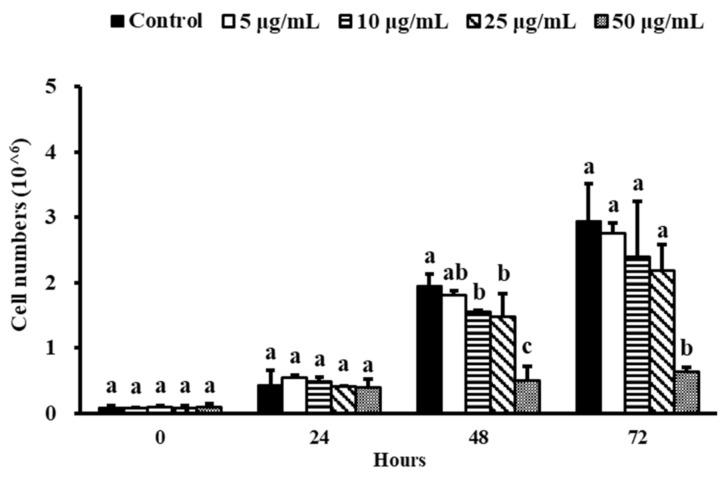
Effects of NEE on cell proliferation in 3T3-L1 preadipocytes. For 72 h, 3T3-L1 preadipocytes were treated or not treated with 5, 10, 25, 50 μg/mL NEE. Duncan’s test was used to determine the significance of the variation in cell proliferation. The results shown are the means and standard deviations (*n* = 3). Significant differences (*p* < 0.05) between the different concentrations of the NEE group are indicated by different letters ^(a–c)^.

For 48 h, NEE at 10, 25, and 50 μg/mL substantially decreased the total cell number of 3T3-L1 preadipocytes (Figure 1). In comparison to the other groups, only 50 μg/mL NEE continued to effectively limit cell growth after 72 h (*p* < 0.05).

### 3.3. Effect of NEE on Cell Viability in 3T3-L1 Preadipocytes

**Figure 2:**
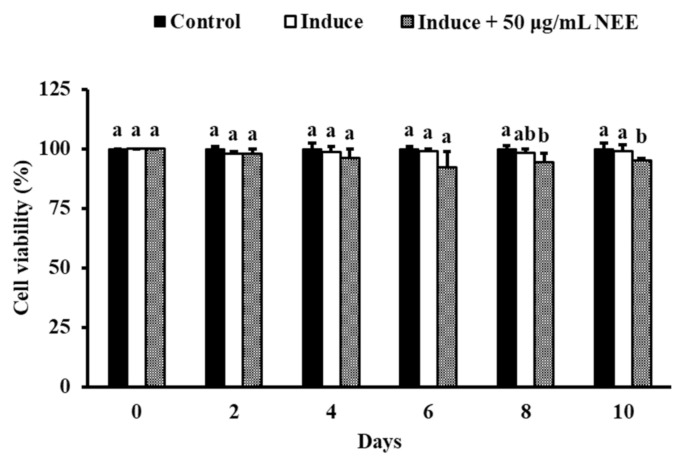
Effects of NEE on cell viability in 3T3-L1 preadipocytes. For 10 days, 3T3-L1 adipocytes were treated or not treated with 50 μg/mL NEE. Cells were harvested every two days, and non-differentiation preadipocytes were the control. Duncan’s test was used to determine the significance of the variation in cell proliferation. The data are the means and standard deviations (*n* = 3). Significant differences (*p* < 0.05) between the different concentrations of the NEE group are indicated by different letters ^(a,b)^.

The NEE at 50 μg/mL significantly decreased cell viability on day 8 and 10 compared with the induced group (*p* < 0.05). However, the percentage of cell viability in all the groups remained more than 95% (Figure 2), which signifies that the NEE exhibited only lower cytotoxicity in 3T3-L1.

### 3.4. Effects of NEE on Lipid Accumulation in 3T3-L1 Preadipocytes

**Figure 3:**
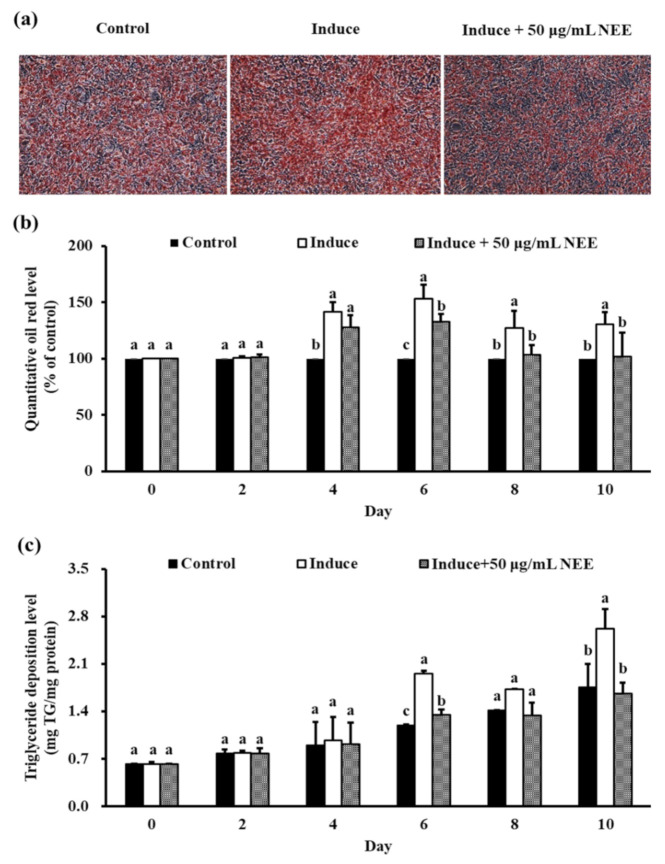
Effects of NEE on lipid accumulation in 3T3-L1 preadipocytes. For 10 days, 3T3-L1 adipocytes were treated or not treated with 50 μg/mL NEE. Cells were harvested every two days. (**a**) Levels of cell lipid accumulation were determined by Oil Red O staining and observation under the microscope (200×) picture only shows the last day. (**b**) The quantity of control lipid accumulation in 3T3-L1 preadipocytes (percent). (**c**) TG concentration (mg TG/mg protein). Control cells are undifferentiated cells. Duncan’s test was used to determine the significance of the variation in cell proliferation. Significant differences (*p* < 0.05) between the different concentrations of the NEE group are indicated by different letters ^(a–c)^.

The differentiation of 3T3-L1 preadipocytes treated with 50 μg/mL NEE reduced the oil red O stanine area (Figure 3a). The level of lipid accumulation and TG deposition significantly decreased from day 6 until day 10, compared with the induced group (*p* < 0.05) (Figure 3b,c).

### 3.5. Effects of NEE on Growth Parameters and Serum Parameters in High Calorie Diet–Induced Obese Rats

The changes in animal body weight of all rats were recorded throughout the 12-week experimental period, and the results are shown in Table 2. The results show that when rats were fed the HD, the weight gain percentage significantly increased, with decreased food intake and FCR, as compared with the ND group (*p* < 0.05). All NEE treatment groups showed significantly decreased weight gain percentage (*p* < 0.05). HNEE significantly increased food intake and FCR in high calorie diet–induced obese rats compared with HD. In the serum parameters, NEE significantly decreased FFA, TC, LDL-C, and glucose AC when compared with the HD group (*p* < 0.05).

### 3.6. Effects of NEE on Animal Characteristics, Body Weight, and Relative Organ Weight in High Calorie Diet–Induced Obese Rat

**Figure 4:**
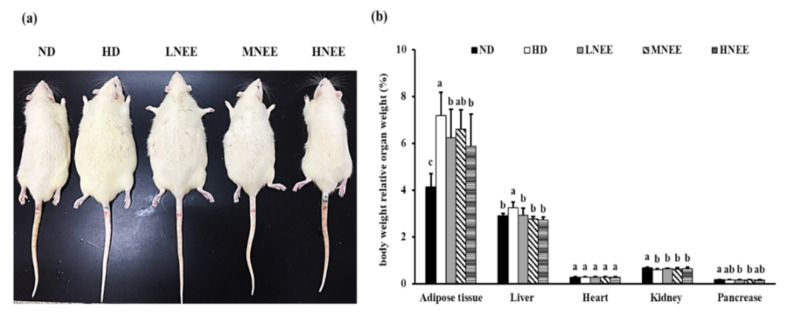
Effects of NEE on animal characteristics and bodyweight-related organ weight in high calorie diet–induced obese rats. Obesity in rats as generated by a high-calorie diet (**a**) and the relationship between body weight and organ weight (**b**). The significance of the organ disparity was determined using Duncan’s test. The means and standard deviations (*n* = 10) are the data. Statistically significant differences between the organs are denoted by different letters ^(a–c)^ (*p* < 0.05). The group names are defined in Table 2 legend.

In Figure 4, we observe the group of HD and LNEE rats had body sizes bigger than the other groups; the obese rat size decreased with the increased NEE dose (Figure 4a). NEE significantly decreased adipose tissue and liver body weight, while relative organ weight remained the same (Figure 4b).

### 3.7. Effects of NEE on Epididymal Adipose Tissue and Liver Histology in High Calorie Diet–Induced Obese Rat

**Figure 5:**
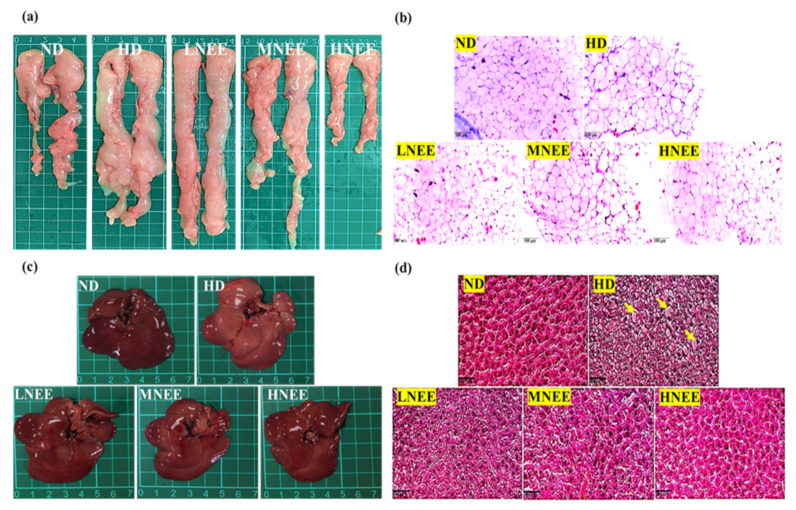
Effects of NEE on epididymal adipose tissue and liver histology in high calorie diet–induced obese rats. Observation of obese rat epididymal adipose tissue (**a**) and liver (**c**), with histological tissue slices in (**b**) and (**d**), respectively, caused by a high-fat diet. Hepatic lipid droplets are shown by the yellow arrowheads (**d**). Hematoxylin and eosin-stained histological tissue slices were examined using a microscope (400×). The group names are defined in Table 2 legend.

The epididymal adipose tissue and liver histology showed HD and LNEE epididymal adipose tissues were bigger than other groups (Figure 5a), and the HD adipocyte size in the histology had the same result. In the MNEE and HNEE epididymal adipose tissue, adipocyte size was evidently smaller than HD or LNEE (Figure 5b). Hepatic lipid droplets are generated in the HD, but NEE ameliorates this situation (Figure 5c,d).

### 3.8. Effects of NEE on Kidney Histological in High Calorie Diet–Induced Obese Rat

**Figure 6:**
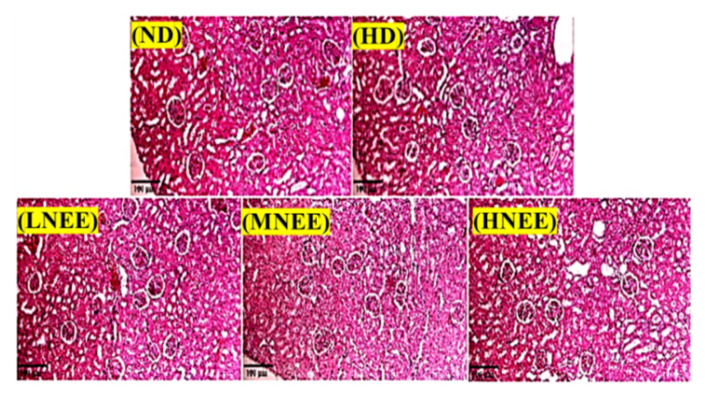
Effects of NEE on kidney histology in high calorie diet–induced obese rats. Observation of obese rat kidney histological tissue slices. Hematoxylin and eosin-stained histological tissue slices were examined using a microscope (400×). The group names are defined in Table 2 legend.

The sections of the kidney were stained using the H&E stain (Figure 6). No changes in the kidney histology were found among the different groups. The results demonstrated the safety of NEE intervention.

### 3.9. Effects of NEE on mRNA Expression of Adipogenesis and Lipid Lysis-Related Gene in Epididymal Adipose Tissue of High Calorie Diet–Induced Obese Rat

**Figure 7:**
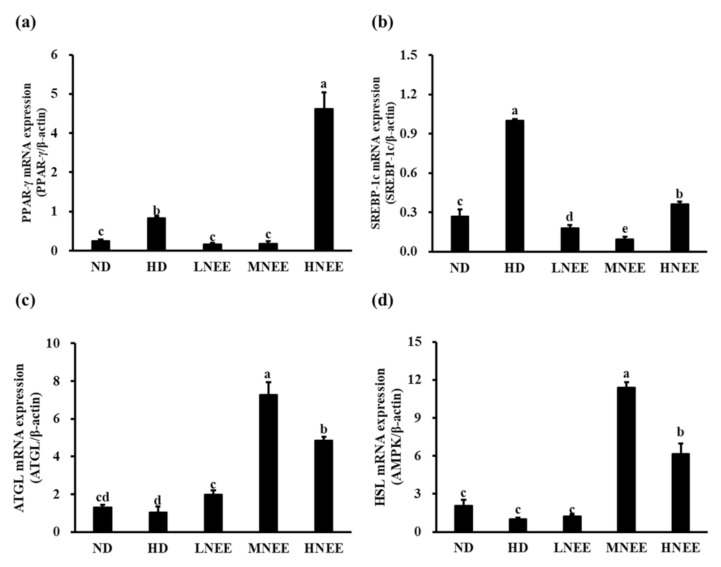
Effects of NEE on mRNA expression of adipogenesis and lipid lysis-related gene in epididymal adipose tissue of high calorie diet–induced obese rats. Quantitative real-time PCR was used to evaluate the mRNA expression of PPAR-γ (**a**), SREBP-1c (**b**), ATGL (**c**), and HSL (**d**). Duncan’s test was used to assess the significance of differences in mRNA expression of adipogenesis and lipid lysis-related genes. The data are the means and standard deviations (*n* = 5). Different letters ^(a–e)^ denote statistically significant differences in mRNA expression (*p* < 0.05). The group names are defined in Table 2 legend.

To better understand the molecular mechanism of NEE’s effects on the adipogenesis (PPAR-, SREBP-1c) and adipolysis genes (ATGL, HSL), mRNA expression was studied in the epididymal adipose tissue of high calorie diet–induced obese rats. In Figure 7, the PPAR-γ gene expression is significantly decreased compared to HD (*p* < 0.05) with LNEE and MNEE, but not HNEE (Figure 7a). In all treatments, SREBP-1c gene expression was significantly lower in the NEE group than in the HD group (Figure 7b). When compared to HD, NEE significantly increased ATGL gene expression (*p* < 0.05) (Figure 7c), while MNEE and HNEE significantly increased HSL gene expression (*p* < 0.05) (Figure 7d).

### 3.10. Effects of NEE on mRNA Expression of the β-Oxidation Related Gene in the Liver of High Calorie Diet–Induced Obese Rats

**Figure 8:**
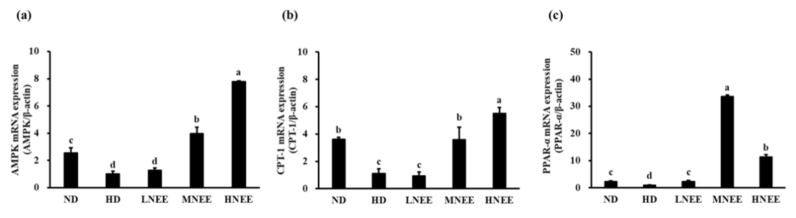
Effects of NEE on mRNA expression of the β-oxidation related gene in the liver of high calorie diet–induced obese rats. Quantitative real-time PCR was used to evaluate the gene expression of AMPK (**a**), CPT-1 (**b**), and PPAR-α (**c**). Duncan’s test was used to determine the significance of differences in mRNA expression of oxidation-associated genes. The data are the means and standard deviations (*n* = 5). Different letters ^(a–d)^ denote statistically significant differences in mRNA expression (*p* < 0.05). The group names are defined in Table 2 legend.

Obesity is largely caused by a malfunction in lipid metabolism. Therefore, we want to understand the molecular mechanism of NEE on mRNA expression of the β-oxidation related gene (AMPK, CPT-1, PPAR-α) in the liver of a high calorie diet–induced obese rat. AMPK and CPT-1 gene expression were considerably higher in MNEE and HNEE than in HD (*p* < 0.05). (Figure 8a,b). When compared to HD, NEE groups substantially enhanced PPAR gene expression in all NEE groups (*p* < 0.05) (Figure 8c).

## 4. Discussion

In recent years, many phytochemicals have been identified in various natural plants, herbs, and algae. Some studies have shown that the secondary metabolites produced in algae tissues are phenolic compounds, flavonoids, sterols, terpenes, and other bioactive compounds, which clearly describe that the different types of polyphenols, flavanols, or terpenoid extracts from algae have anti-obesity effects [36,37,38]. Interestingly, we found the terpenoid compounds are the major phytochemicals: approximately 90% in NEE. Many studies report natural sources of terpenoids for anti-obesity, such as carnosic acid and 14-deoxy-11,12-didehydroandrographolide, isolated from *Andrographis paniculata*, which displays inhibitor adipocyte differentiation by decreasing the gene expression of C/EBPα and PPAR-γ and upregulating mTOR pathways [39].

To our knowledge, this is the first study indicating NEE contains phytochemicals such as polyphenols, flavanols, or terpenoids.

In a number of studies, algae have been shown to reduce lipid accumulation in 3T3-L1 preadipocytes. *Sargassum miyabei* Yendo brown algae ethanol extract suppressed lipid accumulation and differentiation of 3T3-L1 preadipocytes by attenuating the expression of adipogenic- and lipogenic-related genes, such as PPARγ, C/EBPα, C/EBPδ, adiponectin, ATGL, and fatty acid synthase [40]. Siphonaxanthin, a xanthophyll present in green algae, potentially inhibits the adipocyte differentiation and lipid accumulation limited to the early stages of adipogenesis [41]. *Laminaria japonica* has been promoted as a health food in Asia, for which fermentation decreases triglyceride levels, Oil Red O staining, and adipocyte differentiation by reducing the expression levels of C/EBPα/β and PPARγ [42]. *N. commune* lipid extract reduced HepG2 cells SREBP-1 and 2 protein expression, which involve liver cholesterol and triglyceride synthesis [43]. In a previous study, an algae extract was shown to inhibit cell differentiation and lipid accumulation by target signal transduction in cells such as adipocytes and hepatocytes. Our study’s application of NEE decreased 3T3-L1 preadipocyte cell proliferation and lipid accumulation, showing the same results as previous studies. We presume the phytochemicals of NEE led to 3T3-L1 preadipocytes changing the lipogenesis pathway.

Long-term high-calorie food intake leads to body metabolism signaling disorder, lipid deposition in several organs, and changes of body size, serum biochemical parameters, and tissue histology. The findings of this study demonstrate similar characteristics in the high calorie diet–induced obese rat experimental model. An increase of FCR means the intake of food transformed to weight has lower efficiency; our study proved that this induced obesity model is feasible because most obesity develops as a result of abnormal excessive intake of food. Lipase secretes from the pancreas for the digestion of lipids from the diet; hence, we examined serum lipase increases in all treatments with HD and NEE groups, which may have been caused by intake of high-lipid-content food. Phytochemical substances found in algae have been shown to reduce obesity via numerous signaling pathways, including AMPK and PPARs, in certain studies [6,44,45,46]. In an obese mouse model, brown seaweed extract and its derivatives, such as fucoxanthin, reduce obesity by activating AMPK, which also increases oxidation and inhibits lipogenesis [47]. Fucoidan decreases epididymal fat tissue and liver weight and reduces plasma TC and LDL levels, which also restores hepatic lipid droplets in high fat diet–induced obese mice through by down-regulation expression of PPARγ and acetyl CoA carboxylase [48]. L-fucose decreased the expression of PPARγ in high calorie diet–induced obese mice [49]. However, when C57BL/6J mice were fed an AIN-93M diet containing 2.5% or 5% of *Nostoc commune* var. spheroids Kützing (*w*/*w*) for 4 weeks, the plasma TC and TG levels were significantly decreased by increased expressions of gene ACOX-1 and CPT-1α, inhibiting intestinal cholesterol absorption [36,50].

This study shows that NEE has effects on adipogenesis gene expression with low to medium doses only and significantly increases gene expression of AMPK, PPARα, and CPT-1, thereby improving liver lipid metabolism in the obese rats. We also did not find toxic characteristics in treatment with high doses of NEE. These findings show that phytochemicals in NEE from *N. commune* may be responsible for its anti-obesity effect in obese rats fed with a high calorie diet.

## 5. Conclusions

In this study, we show the NEE biological activity, phytochemical compounds, and inhibitory effects of NEE on anti-obesity by inhibitor lipogenesis factor and enhancement of lipolysis and β-oxidation ability. We believe NEE has the potential to be developed as an anti-obesity agent. In the future, we would like to determine the key anti-obesity phytochemical compound in NEE using an in-depth study of NEE in improving metabolism syndrome. Our current research shows that NEE has anti-obesity potential, but to state whether it can be used in the human body requires further investigations or clinical experiments to confirm.

## Figures and Tables

**Table 1:** Quantitative analysis of NEE major phytochemical compounds.

Total Polyphenols (GAE µg/NEE mg)	Total Flavonoids (RUT µg/NEE mg)	Total Terpenoids (OAH µg/NEE mg)
25.89 ± 1.18	19.32 ± 0.45	926.53 ± 0.03

Data are expressed as means ± SD (*n* = 3). *Nostoc commune* ethanol extract (NEE); Gallic acid (GAE); Rutin (RUT); Oleanolic acid hydrate (OAH).

**Table 2:** Effects of NEE on growth parameters and serum parameters in high calorie diet–induced obese rats.

	ND			HD			LNEE			MNEE			HNEE		
*Growth parameters*															
Food intake (g/rat/day)	30.39	±	1.58 ^a^	22.41	±	2.00 ^c^	23.59	±	1.59 ^c^	22.68	±	1.23 ^c^	26.36	±	5.26 ^b^
FCR (%)	8.53	±	0.73 ^a^	5.34	±	0.75 ^c^	5.80	±	0.54 ^c^	5.95	±	0.93 ^c^	7.09	±	1.55 ^b^
Weight gain (%)	136.20	±	9.00 ^b^	166.20	±	20.80 ^a^	148.10	±	14.70 ^b^	139.20	±	21.9 ^b^	144.00	±	17.40 ^b^
*Serum parameters*															
AST (U/L)	99.25	±	3.81 ^a,b^	106.67	±	15.13 ^a^	96.00	±	7.52 ^a^	94.75	±	7.65 ^b^	92.09	±	9.01 ^b^
ALT (U/L)	38.50	±	2.27 ^a^	34.30	±	5.10 ^b^	29.50	±	4.28 ^c^	29.17	±	4.09 ^c^	31.86	±	4.02 ^b,c^
Creatinine (mg/dL)	0.64	±	0.02 ^a^	0.64	±	0.03 ^a^	0.61	±	0.05 ^a,b^	0.59	±	0.03 ^b^	0.59	±	0.03 ^b^
Uric acid (mg/dL)	3.37	±	0.41 ^a^	3.38	±	0.43 ^a^	2.70	±	0.62 ^b^	3.38	±	0.52 ^a^	3.31	±	0.81 ^a,b^
FFA (mmol/L)	1.05	±	0.17 ^b^	1.34	±	0.24 ^a^	1.01	±	0.16 ^b^	1.16	±	0.20 ^b^	1.07	±	0.11 ^b^
Lipase (mmol/L)	26.68	±	4.88 ^d^	42.50	±	6.97 ^c^	47.18	±	12.75 ^c^	66.35	±	9.37 ^b^	78.64	±	2.79 ^a^
TG (mg/dL)	85.00	±	15.78 ^c^	113.14	±	12.51 ^a^	103.33	±	17.92 ^a,b^	98.22	±	14.40 ^a,b,c^	89.83	±	15.47 ^b,c^
TC (mg/dL)	84.33	±	6.91 ^a^	82.17	±	8.77 ^a^	75.50	±	8.43 ^b^	71.31	±	8.97 ^b^	70.70	±	5.21 ^b^
HDL (mg/dL)	60.11	±	4.26 ^a^	58.78	±	8.15 ^a,b^	50.86	±	6.36 ^b^	52.41	±	8.38 ^b^	50.40	±	5.34 ^b^
LDL (mg/dL)	10.79	±	1.58 ^b^	13.33	±	1.87 ^a^	11.50	±	2.20 ^b^	11.00	±	1.93 ^b^	10.92	±	1.98 ^b^
Glucose AC (mg/dL)	128.33	±	14.82 ^b^	165.20	±	15.61 ^a^	94.33	±	12.29 ^b,c^	113.20	±	16.18 ^b,c^	119.00	±	42.83 ^c^

Data are expressed as means ± SD (*n* = 10). Different letters (^a–d^) indicate significant differences among each group (*p* < 0.05). The groups are abbreviated as Normal diet (ND); High calorie diet (HD); HD with low-dose NEE (LNEE); HD with medium-dose NEE (MNEE); HD with high-dose NEE (HNEE).

## Data Availability

Data are available from the corresponding author upon reasonable request.

## Supplementary Materials

The following supporting information can be downloaded at: https://www.mdpi.com/article/10.3390/nu14050968/s1, Table S1: Specific primer of real-time PCR analysis in this study.
