# Supplementary material for: Anti-Obesity Effect of Nostoc commune Ethanol Extract In Vitro and In Vivo
Source: Nutrients. 2022 Feb 24;14(5):968. doi: 10.3390/nu14050968 (PMC8912841; doi:10.3390/nu14050968)
Supplement: Supplementary file 1 [file nutrients-14-00968-s001.zip › nutrients-1575838-supplementary-done.pdf]

nutrients

Supplementary Table S1. Specific primer of real-time PCR analysis in this study.

Gene (rat)	Primer sequence (5′-3′)	Reference (NCBI GenBank)
β-actin	F- AGGCCCCTCTGAACCCTAAG<br>R- CAGCCTGGATGGCTACGTACA	NM031144.3
PPAR-γ	F- AAGTTTGAGTTTGCTGTGAAGTTC.A<br>R- CGATGGGCTTCACGTTCAG	NM001145366.1
SREBP-1c	F- CGGGACAGCTTAGCCTCTACA<br>R- CGGCCACAAGAAGTAGATCA	NM001276707.1
HSL	F-GTCACGCTACATAAAGGCTGCTT<br>R-CAGCCCGATGGAGAGAGTCT	NM012598.1
ATGL	F-GGCCACTGCCATGATGGTA<br>R-GCAGCCACTCCAACAAACG	NM001108509.2
AMPK	F-GTGGATCGCCAAATTATGCA<br>R-AACCTCAGGACCCGCATACA	NM023991.1
CPT-1	F-CCACAAGGCTACAATGGGACAT<br>R-AAGGAATGCAGGTCCACATCA	NM031559.2
PPAR-α	F-GCCCTCGAACTGGATGACA<br>R-CCCTCCTGCAACTTCTCAATG	NM013196.1

F: Forward primer. R: Reverse primer.
